# Infection prevention control in practice: a survey of healthcare professionals' knowledge and experiences

**DOI:** 10.1016/j.infpip.2024.100357

**Published:** 2024-03-09

**Authors:** Isabella Centeleghe, Philip Norville, Jean-Yves Maillard, Louise Hughes

**Affiliations:** a School of Pharmacy and Pharmaceutical Sciences, Cardiff University, Cardiff, UK; b GAMA Healthcare, Hemel Hempstead, UK

**Keywords:** Cleaning, Disinfection, Biofilms, Healthcare professionals

## Abstract

**Background:**

Laboratory experiments are crucial in understanding efficacy of disinfectant products, but without compliance and appropriate application, the effectiveness of products is compromised. This study aims to understand current perceptions and knowledge of healthcare professionals (HCPs) to common cleaning and disinfection routines and microbial contamination, including biofilms, in healthcare environments.

**Methods:**

An online survey, including open and closed questions, was developed. Non-probability convenience and purposive sampling were used: those currently or previously in a healthcare profession were eligible. Survey responses were taken over 24 months, including the COVID-19 pandemic.

**Discussion:**

137 participants completed the survey; over 50% were nurses. Surface cleaning frequency increased post COVID-19 from ‘twice a day’ to ‘three/more times a day’. Disinfection frequency reduced from ‘between every patient’ before COVID-19 to ‘twice a day’ afterwards. A multimethod approach to cleaning and disinfection (70.8%) was predominant when considering the best method to deliver infection control. Most areas of clinical settings were identified as high risk (13/19). Most (87.6%) participants had heard the term ‘biofilm’, mainly at conference/study days (60%). 39.1% said they were aware of dry surface biofilms (DSB) in the healthcare environment.

**Conclusions:**

There remain mixed views on surface cleaning and disinfection within healthcare. Education is important for understanding microbial contamination and tackling problems. More people than expected had heard the term DSB. Infection control practices seemed consistent across responses, however whether this is reality is unknown. This study provides an initial insight into current opinions/knowledge of HCPs and can form basis for further in-depth investigation.

## Introduction

Contaminated surfaces allow for transmission of pathogens throughout the healthcare environment leading to healthcare associated infections (HCAI) [1], one major vector for this is the transmission of pathogens from environmental surfaces and from the hands healthcare workers. Approximately 30–50% of all HCAI are linked to environmental contamination [2]. Ensuring patient safety is crucial and whilst laboratory experiments and testing are essential in establishing the efficacy of disinfectant products, it is also important to focus upon the cleaning-disinfection protocols used in healthcare. National guidelines for cleaning-disinfection do exist, but often these can be imprecise, hard to follow and differ greatly between healthcare facilities [3]. Currently a gap remains in the European and International market for such guidelines [4]. In addition, hospital cleaning-disinfection comes with many challenges, including the process, which products should be used and how to effectively use them [5], which can impact on effectiveness of the infection control and prevention guidelines. Studies have previously shown that inadequate cleaning leaves the areas around a patient bed contaminated, even after they have been deemed as thoroughly cleaned [6,7]. Disinfectant products aim to reduce microbial load, but, if compliance with product preparation and usage and effective pre-cleaning steps are not followed, and workers do not understand the general theory behind disinfection practices, the potential for spread of infection remains important. The general knowledge of healthcare professionals (HCPs) on microbial environmental contamination is therefore a key factor.

The Research Effective Approaches to Cleaning in Hospitals (REACH) training study tested staff members involved in hospital cleaning, on their knowledge and reported practice following implementation of their REACH cleaning bundle. The study found the training bundle helped reduce hospital acquired infection (HAI) throughout Australia and identified an overall increase in understanding of staff to their role and knowledge relating to general cleaning practices [8,9]. In addition, work by Bernstein *et al.* [10], has shown by assessing the knowledge, attitudes, and practices of environmental cleaning alone, environmental service workers could benefit from additional education in order to enhance cleaning practice. The need for an intervention to improve knowledge of HCPs to both cleaning protocols and microbial contamination is still relevant today.

Beyond this, it is important to assess understanding of environmental contamination and potential spread of infection, particularly in association with biofilms. The association of standard microbial biofilms with HCAI has been well documented [11,12], whilst biofilms are notoriously difficult to eliminate [13]. Here, we specifically aim to address HCPs understanding associated with dry surface biofilms (DSB). DSB are biofilms which form on dry environmental hospital surfaces, having been exposed to reduced nutrient sources, a lower water potential and routine cleaning and disinfection processes [14]. DSB are prevalent throughout healthcare facilities and are starting to gain attention due to their resistance to disinfection measures [[15], [16], [17], [18]]. Developing solutions to eradicate DSB will not be effective without of HCPs understanding of DSB, compliance, and correct use of disinfectant products.

To our knowledge, there is no current published research looking at HCPs knowledge of biofilms, with special attention to DSB within healthcare environments. This study aims to identify user perceptions of both cleaning and disinfection to help make future recommendations to cleaning in healthcare, whilst also gaining information on the current knowledge of HCPs to micoorganisms and DSB.

## Methods

### Survey design and recruitment

A survey was developed based on the research question, “what is the knowledge of healthcare professionals to both cleaning and disinfection protocol and environmental microbial contamination?”, focusing on daily routine and cleaning and disinfection methods. Respondents' views on the best intervention methods to prevent spread of infection, the most high-risk areas for transmission of infection and the current methods for measuring cleanliness were sought. Participants were asked questions that would distinguish cleaning from disinfection, which was key to the survey. Finally, participants were asked about their current knowledge of microbes, biofilms, specifically DSB, in the healthcare environment.

Closed answer questions were used in all sections for ease of response, “other” being used when no response fit. Free-format questions were also added to allow participants to expand upon their answers. The survey was formatted using Online Surveys® to maximise accessibility and response. A participant information sheet was prepared and completion of the survey was taken as assumed consent. Ethical approval was given by the Cardiff University School of Pharmacy and Pharmaceutical Sciences Research Ethics Committee (1819-14).

The study population was HCPs. Inclusion criteria were broad; HCPs who are or have previously worked in a healthcare environment. There were no additional exclusion criteria. As such, the survey was reliant on non-probability convenience and purposive sampling [19]. Social media (Twitter) focussed on infection, prevention and control (IPC) specialism groups, was used to purposively target participants. Retweeting was effectively a form of snowball sampling [20]. A gatekeeper was used and the researcher also attended conferences where IPC was the focus. At these, HCPs were provided with the information and invited to participate. The survey was left open for 24 months. This allowed for a maximum opportunity for responses during the SARS-CoV-2 pandemic. This was especially important as there was no opportunity for follow-ups to non-responders as the population was unknown and submission of the survey was anonymous.

### Survey data analysis

Data was extracted from the completed online surveys into Microsoft Excel® and imported into IBM SPSS Statistics v.27® software. Appropriate descriptive statistical analyses were conducted once data had been acquired. Comparative analysis of pre- and post- SARS-CoV-2 responses based on the sector of work was also conducted where appropriate and feasible. ‘Post-pandemic’ was classified as time after and including March 2020, when the UK national lockdown occurred [21]. Those that did not respond or put an answer that was unrecognisable were removed from corresponding data for the questions concerned.

## Results

### Survey response

137 HCPs completed the online survey, 120 were completed pre- SARS-CoV-2 and 17 were completed post. The majority, 78.1%, of participants worked in a hospital – other demographics are shown in Table I.

**Table I:** Respondent demographics pre and post SARS-CoV-2

	Number (pre- SARS-CoV-2)	Number (post-SARS-CoV-2)
**Place of Work (n=137)**		
Hospital	95	12
Community health centre	3	1
GP surgery	2	1
Nursing home	2	–
Residential care home	1	–
Other	17	3
**Job Role (n=136)**		
Nurse	63	8
Doctor	11	–
Midwife	1	–
Consultant	6	1
Clinical Scientist	5	–
Pharmacist	2	–
Academic	2	–
Other	25	12
**Location of Respondent (n=135)**		
Africa	4	–
Asia	5	–
Australia	5	–
Europe	94	17
North America	10	–
**Area of Practice (n=137)**		
A&E (Trauma)	5	–
Cancer	1	–
Care of the Elderly	4	1
General Medicine	4	–
Infection Prevention and Control	70	14
Intensive Care	3	–
Paediatrics	1	–
Psychiatry/Mental Health	2	–
Surgery	6	–
Other	24	2
**Length of Service (n=137)**		
1–5 years	36	6
6–10 years	20	2
11–15 years	14	4
16–20 years	19	2
21+ years	31	3

**Figure 1:**
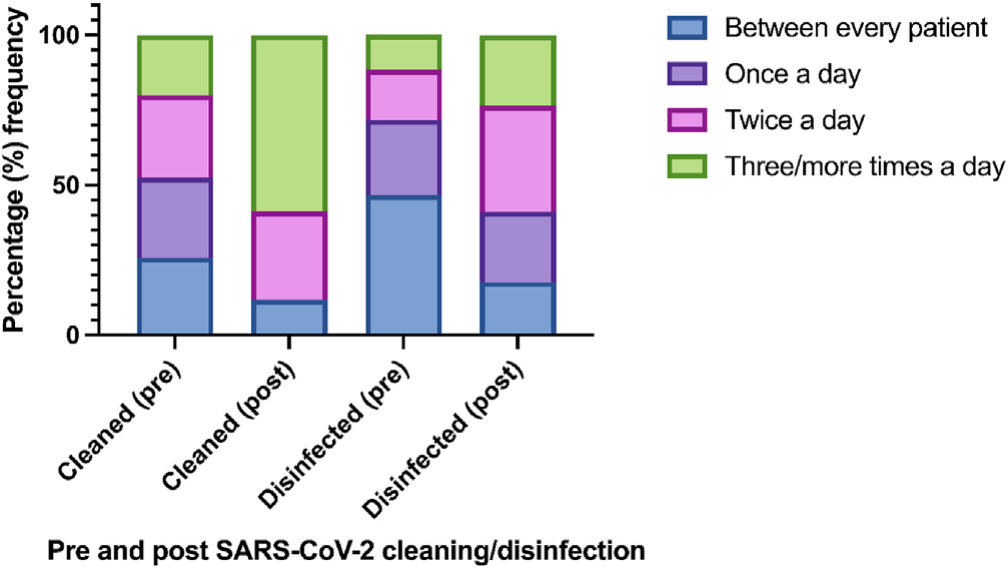
Response data pre- (n=120) and post- (n=17) pandemic to how often healthcare professionals believe hospital surfaces should be cleaned or disinfected on a daily basis.

To understand cleaning and disinfection routine, participants were asked how many times a day they felt hospital surfaces (such as desks and bedside tables etc) should be cleaned or disinfected (Figure 1). Frequency of cleaning three/more times a day was much higher following the pandemic (58.8%) than before (20%). Whereas disinfecting, changed from between every patient (46.7%) pre to twice a day being the most common response post (35.3%) (Figure 1). There was no statistically significant difference between the respondents area of work and their view on the number of times a surface should be cleaned, [X^2^ (15, *N*=137) = 23.284, *P*>0.05], or disinfected [X^2^ (15, *N*=137) = 10.436, *P*>0.05].

**Figure 2:**
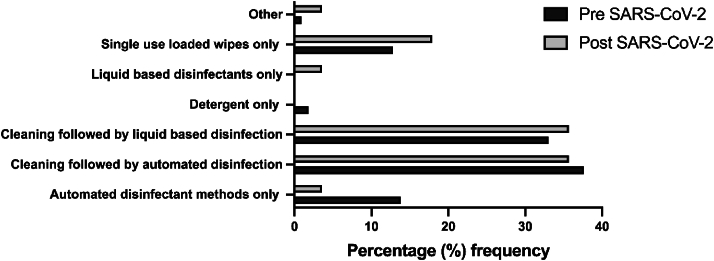
Percentage of both pre- (n=120) and post- (n=17) pandemic responses to what participants believe to be the best method for IPC in a contaminated environment.

Participants were presented with options of various methods to deliver the best infection prevention to a contaminated area. Both pre-and post-pandemic respondents' highest responses were: *Cleaning followed by automated disinfection* and *Cleaning followed by liquid-based disinfection* (Figure 2). Automated disinfection refers to the automated room disinfection systems used to decontaminate hospital rooms. *Cleaning* followed by an additional method were the most frequently chosen methods for IPC (Figure 2). Those few who responded with *Other* (n=2) gave the responses of “*it depends on what the equipment and environment is*”, and *a* “*community deep clean*” was most effective method of delivering IPC.

Survival time of microorganisms on surfaces is essential for companies to consider when creating products for cleaning/disinfection. It is also necessary so that outbreaks can be dealt with effectively. When asked how long various microorganisms can survive (Table II), a couple of respondents answered, “*Don't know”*, while response to bacterial endospore survival was most conclusive out of all microorganisms, with most believing that spores can survive on surfaces for long periods of time (Table II). Over 50% of respondent's post-pandemic thought that viruses can survive on surfaces for days, whereas 30% pre-pandemic had this response (Table II). There was however no statistically significant difference between pre or post SARS-CoV-2 responses and beliefs about survival time of viruses in the environment, X^2^ (6, *N*=137) = 5.604, *P*>0.05.

**Table II:**
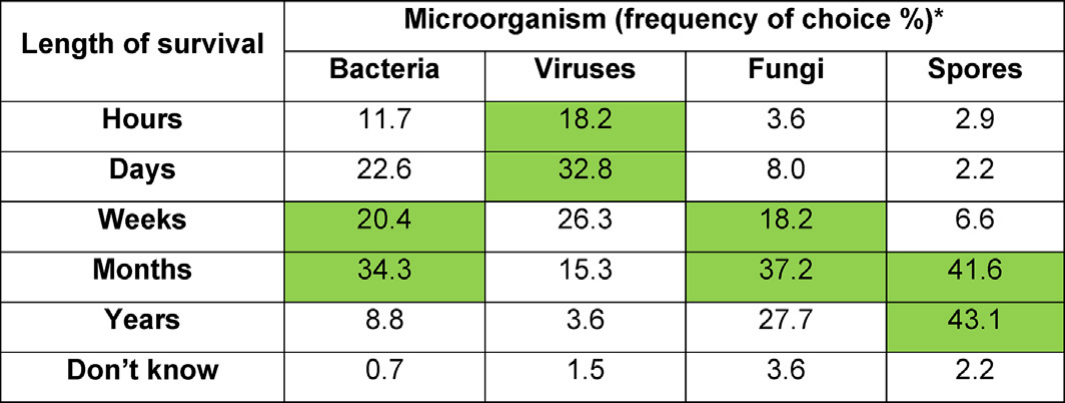
Frequency (%) of all participants' responses regarding their knowledge of microorganism survival. Green cells indicative of average survival time from published data

∗Data from [[22], [23], [24]]

Methods to prevent colonisation of microorganisms is important, participants were given a choice of four different intervention methods and asked which they felt had the greatest impact on IPC. It was unanimous that hand hygiene is believed to be the best intervention method, with 88.3% of all participants selecting this. Vaccinations were least selected with only 1.5% response rate.

Determining microbial contamination of surfaces is important to use alongside cleaning/disinfectant methods to ensure an area is safe and ready for the next patient. The main choices for measuring cleanliness – “*Adenosine Triphosphate (ATP) markers”*, “*Ultraviolet (UV) light”*, “*Culture swabs”* of microorganisms present on surfaces, *indicator products* (stickers, tapes) or *visibly an area looks clean* were given as options to participants alongside *other*. Participants were asked to choose all that they thought applied to the question. Almost half, 48.1%, of 135 participants chose multiple methods; two responses were excluded from the analysis of this question as participants stated “*you cannot measure cleanliness”*. The most common combinations (which included those in multiple methods) were “*ATP markers”* and “*culture swabs”* or “*UV markers”* (21.9%). Out of the 51.9% that only chose a single method, culture swab was the most common answer (20.4%). Participants were then asked specifically which single method is best for measuring cleanliness: “*culture swabs”* was the most popular answer, given by 38.3% of all 133 participants. The answer with the lowest response was indicator products (5.3%).

There are multiple areas in a hospital where there is a risk for transmission and spread of infections. Participants were asked to categorise areas into levels of risk, namely low (green), medium (orange) and high (red) (Table III). Most areas were categorised as high risk by the majority of participants (>55%), with the exception of *nurse station* which received 45.2% medium risk and 45.9% high risk. Only *“café”*, “*clean utility”*, “*floors”* and “*television”* were categorised as low risk areas (Table III).

**Table III:**
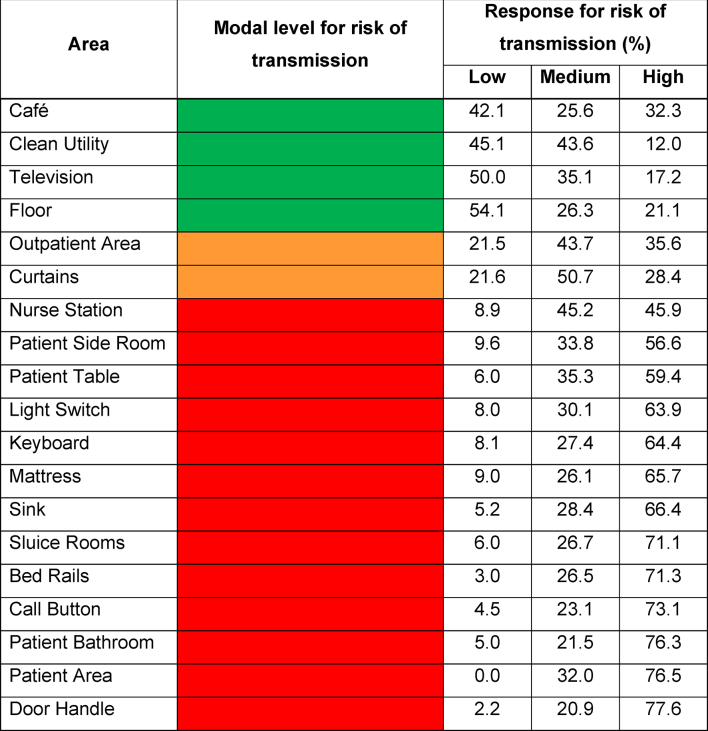
Areas of a hospital and their risk of transmission as rated by participants

The final section of the survey looked to identify current knowledge on biofilms. A total of 87.6% of all participants had heard of the term biofilm, and of these, 83.9% said they knew what the term biofilm means – they were not asked to explain the term. A greater number of participants post-SARS-CoV-2 (94.1%) had heard the term biofilm compared to that pre- SARS-CoV-2 (86.7%). The same can be said for knowledge of the meaning of the term biofilm, where 88.2% of participants post-pandemic understood the term, whereas 83.3% did pre- SARS-CoV-2. There was very little uncertainty over the term biofilm as only 4.4% of all participants were not sure if they had heard the term before and 6.6% were not sure they know what a biofilm is.

We wanted to identify whether there was a relationship between years of experience and having heard the term biofilm, to identify if length of service meant they had more opportunities to be exposed to the word. There was no statistical significance, ((X^2^ (8, *N*=137) = 9.066, *P*>0.05, Pearson Chi-square), between the two suggesting that these two factors are not associated with one another.

However, a statistically significant difference between higher education and knowledge of the term biofilm was determined ((X^2^ (6, *N*=137) = 148.864, *P*<0.001, Pearson Chi-square). Post-hoc z-test and Bonferroni correction with adjusted *P* value of 0.05 outlined the statistically significant difference arose between those that hold a higher education certificate and those that don't in response to both questions and each answer choice (*P*<0.05). It was predicted that most likely those that held a higher education certificate would get the answer correct over those that don't.

*Conference/study days* were the main source of biofilm knowledge (60%), followed by “*scientific journals”* (42.6%), “*talking to other colleagues”* (36.5%) and *“talking to a company rep”* (6.1%); 55.7% of participants chose more than one option. A total of 10/13 participants who chose “*Other”* attained their information from a *“university degree”* – both bachelor's (undergraduate) and postgraduate degrees were mentioned. Only one participant mentioned that their *“own research”* was how they gained information on biofilms.

Participants were asked which types of biofilms they were aware of in relation to health. The vast majority of participants, 90.4%, mentioned more than one biofilm in relation to those that they had come across before. “*Medical device”* (86.1%) and “*drain biofilm”* (76.5%) were the most common answers but less than half (39.1%) of participants had heard the term “*dry surface biofilm”* before. Those who chose *other* indicated “*biofilms on implants”*, and one participant said that “*biofilms will form anywhere*” and so did not make a choice. Participants who did not know, were unsure or had not heard the term biofilm did not respond to this question (16.1%).

## Discussion

HCPs provided an essential insight into the daily cleaning/disinfection routine and knowledge of those in healthcare environments around the UK. Post-pandemic data showed a lean to a higher cleaning and disinfecting frequency per day, especially when considering cleaning. Various factors, including surface material, effect the survival of microbes on a dry surface [25], viruses originating in the respiratory tract such as coxsackie, rhinovirus and influenza are able to survive for a few days [24]. Quantities of published data on the SARS-CoV-2 virus may have influenced choices of both cleaning/disinfection frequency and survival. When considering responses to how long microorganisms survive in the environment, most participants responded with answers in the scale of days and very few stated hours. Thus, cleaning or disinfecting only once a day does not correlate to their current knowledge of survival. This shows that HCPs follow instruction from guidelines, highlighting the importance of a rigorous structure cleaning system.

Results highlighted the importance of cleaning and disinfecting of an area as multimodal. Both pre- and post-pandemic respondents had a unanimous answer of *“cleaning followed by a disinfectant method approach”* was best to deliver IPC effectively. It is well known that a combination of cleaning and disinfection is essential for reducing the threat of HCAI and reducing transmission of pathogens [26]. This study confirms what was also shown with the REACH training programme by Mitchell et al. [8,9], showing participants, in practice, follow the current guidelines for cleaning standards.

Pathogens residing in DSB can survive for prolonged periods in the environment and go unnoticed [14]. Near patient areas, high touch surfaces and high footfall areas were flagged as high-risk areas of transmission. The floor, however, was chosen as a low-risk area. Although floors are deemed low-risk, it is worthwhile noting that they have been found as a reservoir for the transmission of pathogens throughout the hospital environment that readily go unnoticed [27]. It appears areas HCPs believe to be “safe” might pose more threat than first thought, education could help HCPs understand the risks associated with even those termed low-risk areas. Supporting this argument, Houghton et al. [28] reviewed IPC guidelines and adherence of healthcare workers worldwide during the pandemic. They found that when knowledge of IPC was limited, those workers did not adhere to IPC guidelines set out by hospitals, subsequently increasing risk of spreading infection.

Some survey participants chose *“visibly looks clean”* as one of the best methods for measuring cleanliness. This is really important to understand, as they may be relying on other staff members to have completed their job of cleaning/disinfecting an area appropriately, or if they cannot see contamination, they believe a surface to be clean. Highlighting a need for more teaching tools and workshops to understand the science around cleaning and disinfection.

Despite less than half of survey participants knowing what a DSB is, this is surprisingly high considering there are only 33 published articles as of August 2023 on Scopus, the word DSB was first mentioned in a scientific peer-reviewed publication [29]. Whilst respondents had a wide range of length of service, there was no correlation between having heard the term biofilm previously and length of service.

Currently, research is focused on finding a link between HCAI and DSB, but until then, cleaning and disinfectant standards must remain high and perhaps more consistent standards should be put into place as was shown by the spread of responses to how many times a surface should be cleaned/disinfected. This highlights a need to look at both reduction of microbial bioburden on a surface, as well as reduction in transmission post intervention [17]. *“Culture swabs”* was commonly chosen as a method for detection of surface contamination, but we know that DSB cannot be detected through routine swabbing [14], having further implications for patient safety and a clean hospital environment. This could be linked with the two participants that stated, “*You cannot measure cleanliness”*, although they have not stated that is directly linked to DSB it may suggest a similar thought process.

*“Talking to colleagues”* was also amongst the most commonly reported methods of gaining knowledge on infection control topics. Talking to others about current issues and information sharing through social/professional networks are a natural way to provide knowledge to others in the healthcare profession; it shows that word of mouth is essential as an unofficial information tool. In a more formalised expansion of this, HCPs use virtual communities of practice as a tool for continued learning, support, and education [30].

As with most research, this study came with its limitations. Firstly, the main target audience were HCPs, mainly nurses in IPC. As data were being collected during the global pandemic it did become increasingly difficult to get responses. The high proportion of nurses completing the study is likely due to the recruitment approach by researchers. The large quantity of those respondents from an IPC background potentially brings some bias to response. However, this target audience provide the best understanding of current cleaning and disinfection knowledge and practices in the healthcare environment. The questions related to the survival of microorganisms on surfaces required honesty from participants regarding their knowledge, although we assume participants genuinely know from education practices, they may have been inclined just to submit an answer.

Survey analysis has shown that education is really key to tackling problems in the healthcare environment that surrounds IPC. Based on the data presented, this would be best provided through education courses, such as nursing degree, or, through conference and study days. However, an issue that can't be controlled is the veracity of information given when spread via word of mouth putting an emphasis of the importance of education.

## Conclusions

To conclude, we set out to understand the opinions and current knowledge of healthcare professionals on cleaning, disinfection, daily routine and microoganisms. It was clear that there are very mixed views on how often a surface should be decontaminated having implications on infection control in certain healthcare environments. Although infection control methods for both detection and prevention were more consistent across all respondents, there remains the question of if these measures are actually put into practice. This indicates the need for further research and education in the ongoing battle against HCAI.

## Acknowledgements

Thank you to GAMA Healthcare and Cardiff University for funding this project.

## Conflict of interest statement

Phillip Norville is an employee of GAMA Healthcare.

## Funding statement

This work was supported by GAMA Healthcare as a studentship to the lead author and 10.13039/501100000866Cardiff University.

## Ethics statement

Ethical approval was given to the study by the Cardiff University School of Pharmacy and Pharmaceutical Sciences Research Ethics Committee (1819-14).

## References

[1] Weber D.J., Rutala W.A., Miller M.B., Huslage K., Sickbert-Bennett E. (2010). Role of hospital surfaces in the transmission of emerging health care-associated pathogens: norovirus, Clostridium difficile, and Acinetobacter species. Am J Infect Control.

[2] Peters A., Otter J., Moldovan A., Parneix P., Voss A., Pittet D. (2018). Keeping hospitals clean and safe without breaking the bank; Summary of the Healthcare Cleaning Forum 2018. Antimicrob Resist Infect Control.

[3] Castelli A., Norville P., Kiernan M., Maillard J.-Y., Evans S.L. (2022). Review of decontamination protocols for shared non-critical objects in 35 policies of UK NHS acute care organizations. J Hosp Infect.

[4] Assadian O., Harbarth S., Vos M., Knobloch J.K., Asensio A., Widmer A.F. (2021). Practical recommendations for routine cleaning and disinfection procedures in healthcare institutions: a narrative review. J Hosp Infect.

[5] Boyce J.M., Havill N.L., Lipka A., Havill H., Rizvani R. (2010). Variations in Hospital Daily Cleaning Practices. Infect Control Hosp Epidemiol.

[6] Carling P.C., Parry M.F., Von Beheren S.M. (2008). Identifying opportunities to enhance environmental cleaning in 23 acute care hospitals. Infect Control Hosp Epidemiol.

[7] Sitzlar B., Deshpande A., Fertelli D. (2013). An environmental disinfection odyssey: evaluation of sequential interventions to improve disinfection of *Clostridium difficile* isolation rooms. Infect Control Hosp Epidemiol.

[8] Mitchell B.G., White N., Farrington A., Allen M., Page K., Gardner A. (2018). Changes in knowledge and attitudes of hospital environmental services staff: The Researching Effective Approaches to Cleaning in Hospitals (REACH) study. Am J Infect Control.

[9] Mitchell B.G., Hall L., White N., Barnett A.G., Halton K., Paterson D.L. (2019). An environmental cleaning bundle and health-care-associated infections in hospitals (REACH): a multicentre, randomised trial. Lancet Infect Dis.

[10] Bernstein D.A., Salsgiver E., Simon M.S., Greendyke W., Eiras D.P., Ito M. (2016). Understanding Barriers to Optimal Cleaning and Disinfection in Hospitals: A Knowledge, Attitudes, and Practices Survey of Environmental Services Workers. Infect Control Hosp Epidemiol.

[11] Percival S.L., Suleman L., Vuotto C., Donelli G. (2015). Healthcare-associated infections, medical devices and biofilms: risk, tolerance and control. J Med Microbiol.

[12] Haque M., Sartelli M., McKimm J., Bakar M.A. (2018). Health care-associated infections – an overview. Infect Drug Resist.

[13] da Silva R.A.G., Afonina I., Kline K.A. (2021). Eradicating biofilm infections: an update on current and prospective approaches. Curr Opin Microbiol.

[14] Vickery K., Deva A., Jacombs A., Allan J., Valente P., Gosbell I.B. (2012). Presence of biofilm containing viable multiresistant organisms despite terminal cleaning on clinical surfaces in an intensive care unit. J Hosp Infect.

[15] Ledwoch K., Dancer S.J., Otter J.A., Kerr K., Roposte D., Rushton L. (2018). Beware biofilm! Dry biofilms containing bacterial pathogens on multiple healthcare surfaces; a multi-centre study. J Hosp Infect.

[16] Ledwoch K., Said J., Norville P., Maillard J.-Y. (2019). Artificial dry surface biofilm models for testing the efficacy of cleaning and disinfection. Lett Appl Microbiol.

[17] Ledwoch K., Dancer S.J., Otter J.A., Kerr K., Roposte D., Maillard J.-Y. (2021). How dirty is your QWERTY? The risk of healthcare pathogen transmission from computer keyboards. J Hosp Infect.

[18] Ledwoch K., Magoga M., Williams D., Fabbri S., Walsh J., Maillard J.-Y. (2021). Is a reduction in viability enough to determine biofilm susceptibility to a biocide?. Infect Control Hosp Epidemiol.

[19] Babbie E. (2021).

[20] Naderifar M., Goli H., Ghaljaie F. (2017). Snowball Sampling: A Purposeful Method of Sampling in Qualitative Research. Stride Dev Med Educ.

[21] Brown J., Kirk-Wade E., Baker C., Barber S. (2021). https://commonslibrary.parliament.uk/research-briefings/cbp-9068/.

[22] Ulrich N., Nagler K., Laue M., Cockell C.S., Setlow P., Moeller R. (2018). Experimental studies addressing the longevity of *Bacillus subtilis* spores – the first from a 500-year experiment. PLoS One.

[23] Kramer A., Assadian O., Borkow G. (2014). Use of biocidal surfaces for reduction of healthcare acquired infections.

[24] Kramer A., Schwebke I., Kampf G. (2006). How long do nosocomial pathogens persist on inanimate surfaces? A systematic review. BMC Infect Dis.

[25] Jabłońska-Trypuć A., Makuła M., Włodarczyk-Makuła M., Wołejko E., Wydro U., Serra-Majem L. (2022). Inanimate surfaces as a source of hospital infections caused by fungi, bacteria and viruses and particular emphasis on SARS-CoV-2. Int J Environ Res Publ Health.

[26] Weber D.J., Anderson D., Rutala W.A. (2013). The role of the surface environment in healthcare-associated infections. Curr Opin Infect Dis.

[27] Deshpande A., Cadnum J.L., Fertelli D., Sitzlar B., Thota P., Mana T.S. (2017). Are hospital floors an underappreciated reservoir for transmission of health care-associated pathogens?. Am J Infect Control.

[28] Houghton C., Meskell P., Delaney H., Smalle M., Glenton C., Booth A. (2020). Barriers and facilitators to healthcare workers’ adherence with infection prevention and control (IPC) guidelines for respiratory infectious diseases: A rapid qualitative evidence synthesis. Cochrane Database Syst Rev.

[29] Hu H., Johani K., Gosbell I.B., Jacombs A.S.W., Almatroudi A., Whiteley G.S. (2015). Intensive care unit environmental surfaces are contaminated by multidrug-resistant bacteria in biofilms: combined results of conventional culture, pyrosequencing, scanning electron microscopy, and confocal laser microscopy. J Hosp Infect.

[30] McLoughlin C., Patel K.D., O’Callaghan T., Reeves S. (2017). The use of virtual communities of practice to improve interprofessional collaboration and education: findings from an integrated review. J Interprof Care.

