# Supplementary material for: Infection prevention control in practice: a survey of healthcare professionals' knowledge and experiences
Source: Infect Prev Pract. 2024 Mar 9;6(2):100357. doi: 10.1016/j.infpip.2024.100357 (PMC11156693; doi:10.1016/j.infpip.2024.100357)
Supplement: Multimedia component 1 [file mmc1.docx]

**Online survey with four sections, completed by healthcare professionals worldwide. Including questions and multiple-choice answers where appropriate.**

SECTION A: ABOUT YOU

1. Which healthcare setting do you work in?
2. Hospital
3. Residential care home
4. Nursing home
5. GP surgery
6. Community health centre
7. Other please state ………….
8. What is your job title?

Please state………

1. What is your country of residence?

Please state………

1. What is your main area of practice?
2. A&E (Trauma)
3. Cancer
4. Care of the elderly
5. General medicine
6. Intensive Care
7. Paediatrics
8. Psychiatry/mental health
9. Surgery
10. Other please state ………….
11. How long have you been working in your healthcare role?
12. 1-5 years
13. 6-10 years
14. 11-15 years
15. 16-20 years
16. 21+ years
17. Have you attained any specific training/qualifications relation to infection control?

Please state……..

SECTION B: DAILY ROUTINE

1. Out of these methods, which methods are most effective when measuring cleanliness? (Please tick all that apply)
2. Adenosine Triphosphate (ATP) assay
3. Culture of microorganisms from a swab taken
4. Indicator products (stickers/tape)
5. Ultraviolet (UV) markers
6. Visibly it looks clean
7. Other please state ………….
8. From the above, which do you believe is the **single most important** method?
9. Adenosine Triphosphate (ATP) assay
10. Culture of microorganisms from a swab taken
11. Indicator products (stickers/tape)
12. Ultraviolet (UV) markers
13. Visibly it looks clean
14. Other please state ………….
15. In your opinion, which intervention has the greatest impact on infection prevention and control within the healthcare setting?
16. Hand hygiene
17. Outbreak control
18. Surface cleaning
19. Surface cleaning and disinfection
20. Vaccinations
21. Other please state ………….
22. Please tick one out of the three risk factor options for each area/room in the grid, with regards to the transmission and spread of infection in the healthcare setting. For example, high would mean that you believe there is a high chance of spread and transmission of infection in this area/room.

|  | **Risk of transmission** | | |
| --- | --- | --- | --- |
| **Area/room** | Low | Medium | High |
| Café |  |  |  |
| Clean utility |  |  |  |
| Nurse station |  |  |  |
| Outpatient area |  |  |  |
| Patient area |  |  |  |
| Patient bathroom |  |  |  |
| Patient side room |  |  |  |
| Sluice rooms |  |  |  |

1. Please tick one out of the three risk factor options for each item in the grid, with regards to the transmission and spread of infection in the healthcare setting. For example, high would mean that you believe that this item definitely enables the spread and transmission of infection.

|  | **Risk of transmission** | | |
| --- | --- | --- | --- |
| **Item** | **Low** | **Medium** | **High** |
| Bed rails |  |  |  |
| Call button |  |  |  |
| Curtains |  |  |  |
| Door handle |  |  |  |
| Floor |  |  |  |
| Keyboard |  |  |  |
| Light switches |  |  |  |
| Mattress |  |  |  |
| Patient table |  |  |  |
| Sink |  |  |  |
| Television |  |  |  |

SECTION C: CLEANING AND DISINFECTION

1. How often do you think hospital surfaces (e.g. desks, bedside tables) should be **cleaned** on a routinely basis?
2. Between every patient
3. Four/more times a day
4. Less than once a day
5. Once a day
6. Three times a day
7. Twice a day
8. How often do you think hospital surfaces (e.g. desks, bedside tables) should be **disinfected** on a routinely basis?
9. Between every patient
10. Four/more times a day
11. Less than once a day
12. Once a day
13. Three times a day
14. Twice a day
15. Which one of the methods below do you believe delivers the **best** infection prevention in a contaminated area?
16. Automated disinfectant methods only (e.g. UV light, hydrogen peroxide)
17. Cleaning and automated disinfection
18. Cleaning followed by liquid based disinfection
19. Cleaning only (detergent)
20. Liquid based disinfectants only
21. Single use loaded wipes only (antibacterial, sporicidal)
22. Other please state ………….
23. From the following options, typically, how long do you think these microorganisms can survive in the healthcare environment? (Please tick one option for each microorganism)

|  | **Microorganisms** | | | |
| --- | --- | --- | --- | --- |
| **Survival** | Bacteria | Viruses | Fungi | Spores |
| Hours |  |  |  |  |
| Days |  |  |  |  |
| Weeks |  |  |  |  |
| Months |  |  |  |  |
| Years |  |  |  |  |
| Don’t know |  |  |  |  |

SECTION D:

1. Have you **heard** the term “microbial biofilms”?
2. Yes
3. No
4. Not sure
5. Do you know what a microbial biofilm is?
6. Yes
7. No
8. Not sure

If YES, please continue to question 3. If NO or NOT SURE, thank you for your time in completing the survey.

1. Where did you gain this information on microbial biofilms? (Please tick all that apply)
2. Conference/study day
3. General press (e.g. Newspapers)
4. Online
5. Professional magazine (e.g. Nursing Times)
6. Scientific journal (e.g. Journal of Clinical Microbiology, Nature)
7. Short workshop/training session
8. Social media (e.g. Twitter)
9. Talking to colleagues
10. Talking to a company rep
11. Other please state ………….
12. What type of biofilms are you aware of? (Please tick all which apply)
13. Dental
14. Drain
15. Dry surface
16. Medical device
17. Wet
18. Other please state ………….

Thank you for taking the time to complete the survey. If you have anything else you want to mention on the areas touched upon, please could you state below.
